# Histone acetyltransferases and histone deacetylases in B- and T-cell development, physiology and malignancy

**DOI:** 10.18632/genesandcancer.65

**Published:** 2015-05

**Authors:** Leila Haery, Ryan C. Thompson, Thomas D. Gilmore

**Affiliations:** ^1^ Department of Biology, Boston University, Boston, MA, USA

**Keywords:** HAT, HDAC, acetylation, B cells, T cells

## Abstract

The development of B and T cells from hematopoietic precursors and the regulation of the functions of these immune cells are complex processes that involve highly regulated signaling pathways and transcriptional control. The signaling pathways and gene expression patterns that give rise to these developmental processes are coordinated, in part, by two opposing classes of broad-based enzymatic regulators: histone acetyltransferases (HATs) and histone deacetylases (HDACs). HATs and HDACs can modulate gene transcription by altering histone acetylation to modify chromatin structure, and by regulating the activity of non-histone substrates, including an array of immune-cell transcription factors. In addition to their role in normal B and T cells, dysregulation of HAT and HDAC activity is associated with a variety of B- and T-cell malignancies. In this review, we describe the roles of HATs and HDACs in normal B- and T-cell physiology, describe mutations and dysregulation of HATs and HDACs that are implicated lymphoma and leukemia, and discuss HAT and HDAC inhibitors that have been explored as treatment options for leukemias and lymphomas.

## INTRODUCTION

B and T cells have a variety of cellular subtypes that arise through a complex series of developmental events. The function of these various immune cell subtypes can be altered by numerous extracellular factors, including antigens, cytokines, and growth factors. Many of these developmental and functional processes are controlled by large-scale changes in gene expression, either due to epigenetic changes in chromatin structure or to the activity of specific transcription factors (TFs). Histone acetyltransferases (HATs) and histone deacetylases (HDACs) are two opposing classes of enzymes that play widespread roles in regulating transcription either by altering chromatin structure or by modulating the activity of specific TFs. Thus, it is perhaps not surprising that HATs and HDACs play roles in maintaining hematopoietic precursors and in coordinating their maturation into various subtypes of B and T cells.

As with many proteins that have important roles in normal developmental and cell-specific proliferation and survival processes, HAT and HDAC activity is altered in many B- and T-cell malignancies. Moreover, several HDAC inhibitors (HDACi) have been found to reduce the proliferation of B and T cancer cells *in vitro* and *in vivo*. As an outcome of such basic research, there are four FDA-approved HDACi being used clinically to treat T-cell lymphoma and multiple myeloma, and there are several clinical trials using HDACi for the treatment of B- and T-cell cancers.

In this review, we describe the roles of HATs and HDACs in normal B- and T-cell development and function, and also discuss alterations in HAT/HDAC activity in B- and T-cell malignancies. Finally, we summarize the current status of HAT and HDAC inhibitors as potential therapies for cancers affecting B and T cells.

### Overview of the Regulation of Transcription by HATs and HDACs

HATs and HDACs carry out acetylation and deacetylation, respectively, of the ε-amino group of specific lysine residues on target proteins. The addition of an acetyl group prevents the formation of positive charges on the lysine amino group, and thus, can affect protein activity. Through this reversible catalytic event, HATs and HDACs can regulate transcription in two general ways: 1) by altering histone acetylation patterns, thereby modulating chromatin structure and its accessibility to transcriptional regulatory proteins [1, 2], and 2) by acetylating and affecting the activity of non-histone substrates that directly regulate transcription, including a diverse array of TFs [3].

HATs are a subtype of transcriptional coactivators, in that they possess intrinsic acetyltransferase activity and can enhance the ability of a TF to activate transcription. In general, HAT-mediated acetylation of nucleosomal histones increases the accessibility of DNA to TFs and leads to increased transcription at a given DNA locus. Acetylation of specific TFs by HATs can also increase their ability to bind DNA, resist proteasomal degradation, or interact with other TFs or coactivators, and consequently, direct acetylation of TFs can also be a transcriptional activating event [3]. In addition, by serving as protein scaffolds, HATs can promote the formation of transcriptional activating complexes near a gene promoter. This scaffolding function does not necessarily require HAT enzymatic activity, but rather is defined by the protein-interaction domains of these relatively large molecules.

HDACs, on the other hand, generally act as transcriptional corepressors by deacetylating nucleosomal histones, which can lead to chromosomal condensation and the exclusion of transcriptional activating complexes. Additionally, large HDAC-containing repressor complexes can localize to specific gene loci and exclude activating molecules, including HATs, from interacting with TFs. HDACs can also deacetylate specific TFs, decreasing their DNA-binding activity, inducing their degradation, or changing their subcellular localization or protein-protein interactions [4].

### Families of Human HATs and HDACs

#### HAT families

**Figure 1:**
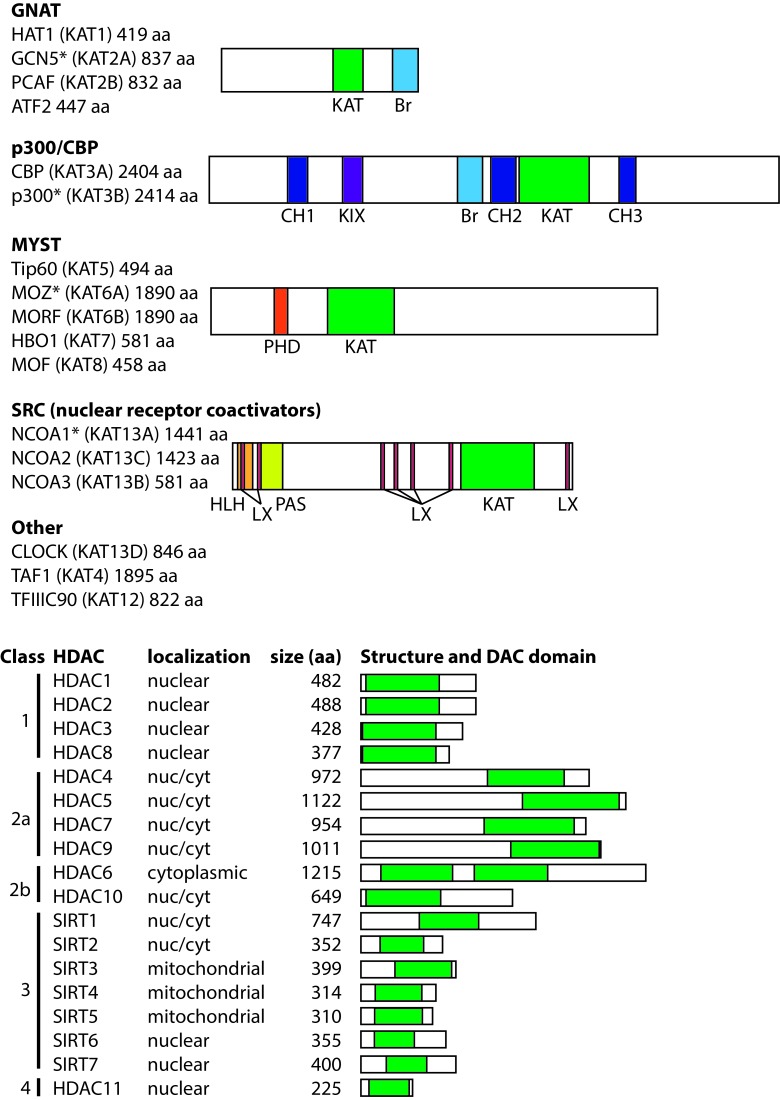
The general structures of human HATs and HDACs Schematic representations are drawn approximately to scale. The catalytically active domains and other conserved domains are shown: acetyltransferase domain (KAT), bromodomain (Br), cysteine/histidine domain (CH), KIX domain, PH-D finger (PHD), helix-loop-helix domain (HLH), LXXLL motif (LX), PAS domain (PAS). All human HATs are listed with the gene alias in parentheses. Size of each HAT is shown as number of amino acids. A representative HAT (indicated by asterisk) is shown for each family. All HDACs are listed with their predominant subcellular localizations; those that shuttle between the nucleus and cytoplasm are indicated as nuc/cyt. Catalytic domains are indicated with green boxes.

There are 17 human HATs, which are divided into five families based primarily on the extent of sequence similarity [5] (Figure 1). Although HATs can act on a broad range of substrates *in vitro*, HATs are usually directed to specific targets *in vivo*, and thus, HAT families generally have distinct biological functions. The non-catalytic domains of HATs are responsible for dictating this substrate specificity, and HAT families generally have conserved protein-protein interaction and reader domains (e.g., bromodomains, PHD fingers), which enable them to localize to particular genomic sites and recognize specific chemical or epigenetic modifications. The size of the catalytic HAT domain and the mechanism of catalysis also differ between HAT families. For example, CBP and p300 utilize a “hit and run” kinetic model defined by an initial binding of acetyl-CoA followed by transient binding to the target lysine [6, 7], whereas the GNAT family HATs adopt a ternary complex during catalysis [8].

GNAT family HATs (GCN5, HAT1, PCAF, ATF2) are generally part of large, multi-protein complexes that contain TBP-associated factors (TAFs) and a single catalytic HAT subunit (reviewed in [9]). Two well-characterized complexes found in humans are the 700 kDa (i.e., ATAC) and 2 MDa (i.e., TFTC, STAGA, and PCAF) complexes. These large HAT-containing complexes play roles in global chromatin acetylation (i.e., the deposition of acetyl marks on histones) and as coactivators of genes when recruited to DNA by specific TFs or regulatory proteins. Members of the GNAT family, especially PCAF, also acetylate specific TFs and modulate their activity (e.g., p53, BRCA2, PTEN). GNAT family members have a conserved C-terminal bromodomain, which has been shown to be an acetyl-lysine targeting motif. GNAT family member ATF2 is the only sequence-specific DNA-binding transcriptional activator with intrinsic HAT activity.

The CBP/p300 HATs are large (~300 kDa), highly related proteins with a single HAT domain, a bromodomain, and several cysteine–histidine-rich (CH) domains that participate in a variety of protein-protein interactions [5]. Indeed, CBP/p300 have been shown to acetylate over 75 target proteins, including all histone proteins, as well as numerous TFs. By virtue of their multiple protein-protein interaction domains, CBP/p300 can also promote transcriptional activation by nucleating transcriptional complexes at promoters in a non-enzymatic manner. Although they generally act as coactivators, in some cases, CBP/p300 appear to be involved in transcriptional repression [10].

The MYST family of HATs (TIP60, MOZ, MORF, HBO1, MOF) is characterized by a conserved MYST domain that contains the catalytically active HAT domain. The two largest family members, MOZ and MORF, also have a PHD zinc finger domain, which recognizes methyl-lysine-containing motifs [11], and a C-terminal transactivation domain that interacts with various transcription factors, including hematopoietic cell regulators PU.1 and Runx1 [12-14]. Most MYST family HATs act as catalytic subunits of large multiprotein complexes, including the ING family of tumor suppressors [15].

The steroid receptor coactivators (SRCs) include three HATs (NCOA1, NCOA2, NCOA3) that enhance transcription of genes responsive to liganded nuclear receptors [16]. In addition to the HAT domain, SRCs contain three conserved domains: 1) an N-terminal bHLH-PAS (basic helix-loop-helix-Per/ARNT/Sim), which is necessary for interaction with other coactivators; 2) one or more LXXL repeats, which mediate interactions with other nuclear receptors and cofactors; and 3) two C-terminal transcriptional activation domains (AD1 and AD2). Although SRCs have been associated with various human cancers, currently they are not known to have a role in hematopoiesis or B-/T-cell function.

Other HATs that are not clearly part of a family include the following; TAF1 (TAFII250), a subunit of the TFIID general TF complex; CLOCK, which is primarily involved in circadian rhythm; and the 90 kDa subunit of TFIIIC, which is involved in the control of general transcription in a complex with RNA polymerase III.

#### HDAC families

To counterbalance the impact of HATs on protein function and genome structure, there are 18 human HDACs, which are commonly divided into four major classes based on homology to yeast orthologs (Figure 1): class 1 (HDAC1, 2, 3 and 8), class 2 (HDAC4, 5, 6, 7, 9, and 10), class 3 (aka sirtuins; SIRT1, 2, 3, 4, 5, 6, and 7), and class 4 (HDAC11) [17-20]. HDAC classes differ in their structure, substrate specificity, enzymatic mechanism, subcellular localization, and tissue-specific expression. Even though most HDACs contain a nuclear localization signal (NLS) and, in some cases, a nuclear export signal (NES), HDACs often localize to specific subcellular regions due to protein-protein interactions with proteins that direct their cellular localization.

The “classical” HDACs are those in classes 1, 2a, 2b, and 4, and they have a conserved ~390 aa catalytic domain and Zn2+-dependent deacetylase activity. The conserved, ~275 aa catalytic domain of the class 3 sirtuins is NAD+-dependent and unrelated to the catalytic domain of the classical HDACs [21, 22]. These differences in their catalytic mechanisms have implications for inhibition of HDAC activity, and thus, many of the HDAC inhibitors (HDACi) used in cancer therapeutics target the classical HDACs (discussed below).

Class 1 HDACs are ubiquitously expressed and localize almost exclusively to the nucleus. The class 2 HDACs are generally much larger than class 1 HDACs, show tissue-specific expression patterns, and often shuttle in and out of the nucleus. In general, HDACs in both classes 1 and 2 are found in large transcriptional repressing complexes, and are recruited to DNA either by other proteins in those complexes or by other DNA-binding proteins. These large protein complexes play roles in HDAC localization and substrate specificity, can act as scaffolds to recruit DNA-binding proteins, and provide the cofactors required for HDAC function. Indeed, lack of these cofactors limits the activity of some recombinant HDACs [23]. HDAC 11 is the only class 4 HDAC, and although it shows sequence similarity to class 1 and 2 HDACs, it does not exist within any of the known HDAC complexes.

Class 3 sirtuins vary in their subcellular localization and interact with a diverse array of TFs and other, primarily non-histone, substrates [24]. For example, SIRT1 can directly interact with substrates involved in the stress response, including p53, FOXO proteins, and NF-κB. The mitochondrially-localized sirtuins (SIRT3, 4, 5) can regulate mitochondrial function, respiration, and energy consumption [25]. Some sirtuins (SIRT4 and 6) lack deacetylase activity, but possess ADP-ribosyl-transferase activity and play roles in metabolism and DNA repair.

### HATs and HDACs in B- and T-cell Development

#### HATs and HDACs in early hematopoietic development

B- and T-cell development involves controlled stages of gene expression programs and genomic instability, which ultimately give rise to the diversity of cells that provide adaptive immunity. These developmental stages are tightly regulated by a large variety of TFs and are coupled with the accessibility of DNA to factors that coordinate chromosomal rearrangements. HATs and HDACs play major roles in normal B- and T-cell development because they can interact with hematopoietic regulators and TFs, as well as affect DNA accessibility by modifying chromatin structure near relevant target genes. In this section, some roles of HATs and HDACs in normal hematopoiesis, lymphopoiesis, and B- and T-cell function are discussed (summarized in Table 1).

Much of what is known about the role of HATs and HDACs in the development of mammalian B and T cells comes from the study of whole knockout (KO) mice and of mice with tissue-specific inactivation of individual HATs and HDACs. In most cases, whole-mouse HAT and HDAC KOs are embryonic lethal. Therefore, to explore the roles of these HATs/HDACs in hematopoiesis, either hematopoietic progenitors have been isolated from KO mice or hematopoietic lineage-specific gene KOs have been generated.

Mice with cell-specific KOs of CBP or p300 have defects in maintenance and differentiation of hematopoietic stems cells (HSCs) [26, 27]. The defect in hematopoiesis in p300-null stem cell lines can be rescued by re-expression of wild-type p300 or when an extra copy of CBP is placed under control of the p300 locus, suggesting that the total dosage of HAT activity by CBP/p300 is critical for hematopoietic maintenance and differentiation rather than the specific activity of either individual HAT [26, 27]. Likewise, in the MYST family, whole animal KO of MOZ is embryonic or perinatal lethal, and MOZ KO embryos show a dramatic reduction in the number and repopulation capacity of hematopoietic progenitors, whereas mice with heterozygous KO or with a HAT deletion of MOZ often show intermediate phenotypes, suggesting a dose-dependent requirement for activity of this HAT [13, 28].

**Table 1:** Roles of HATs and HDACs in B- and T-cell development and function

HAT or HDAC	Role in early hematopoiesis	Role in B cell	Role in T cell
GCN5		Regulates transcription of IgM H-chain. Activates IRF4 (Required for B-cell differentiation)	
PCAF		HSC maintenance (via TRAPP), acetylates E2A (major role in B-cell differentiation)	Positively regulates FOXP3 expression
CBP	HSC maintenance	Development of peripheral B cells	
p300	HSC maintenance	Development of peripheral B cells	Regulates CCR9 expression during thymocyte migration. Acetylates FOXP3, which is required for survival of Tregs
TIP60	HSC maintenance		Acetylates FOXP3, and is important for survival of Tregs
MOZ	HSC maintenance	Enhances HOXA9 expression, activates PU.1	
HBO1			Regulates CD4/CD8 expression patterns in thymocytes. Activates CD8 expression via global H3K14 acetylation
MOF	HSC maintenance and expression of hematopoietic regulators		
HDAC1	HSC maintenance. ERK1/2 repression via SMAD1/5.		No effect on T-cell development, but leads to upregulation of HDAC2. Represses cytokine production (IL-4, IL-5, IL-10) in activated T cells and during T effector cell differentiation. Negatively regulates FOXP3 expression.
HDAC2	HSC maintenance		
HDAC3	Repopulation of B and T cells and HSC self-renewal		Deacetylates FoxP3, which reduces Treg development and suppressive function
HDAC4		Deacetylates BCL6, activating genes (lymphocyte activation, differentiation, apoptosis)	
HDAC5		Phosphorylated by protein kinases D1 and D3 and exported as a result of BCR signaling. Deacetylates BCL6, which activates genes for lymphocyte activation, differentiation, and apoptosis	
HDAC7		Phosphorylated by protein kinases D1 and D3 and exported as a result of BCR signaling. Deacetylates BCL6, which activates genes for lymphocyte activation, differentiation, and apoptosis	Represses Nur77 expression during TCR negative selection. Regulates gene expression during TCR positive selection
HDAC9			Deacetylates FoxP3, which reduces Treg development and immunosuppressive activity
HDAC6			Controls IgM and IgG levels upon antigen stimulation. T-cell migration. Immune synapse formation. Deacetylates FOXP3, which reduces Treg development and immunosuppressive activity
HDAC11			Represses IL-10 expression in APCs
SIRT1			Deacetylates FoxP3, which reduces Treg cell development and immunosuppressive activity

To study the role of specific p300 domains in hematopoiesis, a series of p300 deletion mutants were re-expressed in p300-null embryonic stem cells (ESCs), and their ability to contribute to hematopoiesis was analyzed. These studies showed that p300 mutants lacking the KIX or CH1 domain had reduced abilities to induce hematopoiesis, and these defects were similar to the parental p300-null cells [27]. This reduction in hematopoiesis is thought to be due to an inability of KIX and CH1 deletion mutants to interact with the TF MYB [27, 29]. Interestingly, the presence of a functional HAT domain in p300 appears to play a role in limiting the proliferation of hematopoietic precursors, in that expression of a HAT-deficient p300 mutant in p300-null cells leads to increased numbers of hematopoietic cell populations, as compared to re-expression of wild-type p300 [27]. The dispensability of the CBP/p300 HAT domain for hematopoiesis may be due to their interaction with the HAT PCAF, which provides catalytic HAT activity in some CBP/p300 signaling contexts (e.g., myogenic differentiation) [30]. Thus, unlike the MYST family, which relies on HAT activity for proper proliferation of HSCs (described below), the HAT domains of CBP/p300 may actually reduce the proliferation of some hematopoietic cell types.

Among the MYST family proteins, the role of MOZ in hematopoiesis was determined by analyzing the hematopoietic progenitors in MOZ KO mice. Whole-mouse MOZ KO reduces the number of HSCs, and also affects the ability of these stem cells to renew and reconstitute the hematopoietic system [13]. HSCs from MOZ KO mice have reduced HOXA9 expression, which is known to reduce the differentiation potential of HSCs. MOZ is also a transcriptional coactivator of PU.1, and reduced PU.1 activity can explain many of the phenotypes seen in MOZ KO mice [13, 31, 32]. In mice expressing HAT-deficient MOZ, hematopoietic progenitors are severely defective in competitive repopulation assays, demonstrating a critical role of the MOZ catalytic domain in HSC functionality [33]. These defects were linked to a marked deficiency in the proliferative capacity of HAT-deficient MOZ precursors [33]. Loss of MYST family member MOF in ESCs is associated with a reduction in the expression of some hematopoietic genes, suggesting a role for MOF in hematopoiesis [34]. Additionally, conditional KO of the coactivator TRRAP, which can act as a subunit of TIP60 and PCAF HAT-containing complexes, leads to loss of HSCs [35].

HDAC1 and 2 have overlapping critical roles in early hematopoiesis and HSC homeostasis, largely by acting in a SIN3A/HDAC1/2-repressor complex. While mice with bone marrow-specific deletions of either HDAC1 or HDAC2 show only moderate phenotypes, the simultaneous deletion of HDAC1 and HDAC2 (or SIN3A alone) leads to nearly complete loss of hematopoietic progenitors, causing severe reduction in the numbers of spleen, thymic and bone marrow cells [36-38]. During HSC emergence in zebrafish, HDAC1 is recruited to the *erk* promoter by SMAD1/5, and represses *erk1/2* expression by deacetylating H3K9 and H3K27 [39]. Conditional KO studies have shown that HDAC3 is required for DNA replication in HSCs, which is essential for their ability to produce B- and T-cell progenitors [40].

#### HATs and HDACs in B-cell development and function

Disruption of p300 or CBP at the pro-B cell stage results in a 25-50% reduction in the number of B cells in the peripheral blood; however, the number of pro-B, pre-B, and immature B cells in the bone marrow is unaffected [41]. Loss of CBP at this stage does not drastically perturb gene expression in resting B cells, as ~99% of microarray transcripts measured in CBP-null cells were within 1.7-fold of controls [41]. These results indicate that loss of either p300 or CBP starting at the pro-B cell stage is not required for B-cell function, possibly due to functional redundancy of these two HATs. In contrast to the single KOs, the double KO of CBP and p300 in pro-B cells causes a dramatic reduction in the number of peripheral B cells [41].

With the exception of mature B cells, the HAT activity of MOZ is required for the cell proliferation required to maintain healthy numbers of hematopoietic precursors. That is, mice expressing a HAT-deficient MOZ protein show an approximately 50% reduction in the numbers of pro/pre-B cells and immature B cells, whereas the number of mature B cells and their ability to carry out antibody responses is unaffected [33].

KO of GCN5 in the chicken immature B-cell line DT40 showed that GCN5 regulates transcription of the IgM H-chain gene, and GCN5 deficiency decreased membrane-bound and secreted forms of IgM proteins [42]. GCN5 also directly activates expression of the TF IRF4, which is required for B-cell differentiation [43]. PCAF acetylates the TF E2A, which plays a major role in the differentiation of B lymphocytes [44].

HDACs also appear to play a role in signaling from the B-cell receptor (BCR). During BCR activation, HDACs 5 and 7 are phosphorylated by protein kinases D1 and D3 and exported from the nucleus, suggesting a link between BCR function and epigenetic regulation of chromatin structure [45].

A major regulator of B-cell differentiation is the TF BCL6, which represses a set of target genes during proper germinal center (GC) development [46]. BCL6 also serves as an anti-apoptotic factor during an immune response, which enables DNA-remodeling processes to occur without eliciting an apoptotic DNA damage response [47, 48]. To achieve GC-specific gene expression, BCL6 is recruited to a large repressor complex that contains HDAC4, 5, and 7, and localizes to the nucleus to regulate its target genes [49]. Treatment of cells with an HDACi results in hyper-acetylation of BCL6, which derepresses expression of BCL6 target genes involved in lymphocyte activation, differentiation, and apoptosis [50, 51].

In B cells, HDAC1 and 2 play a key, redundant role in cell proliferation and at certain stages of development. That is, in early B cells the combined KO of HDAC1 and 2 results in a loss of further B-cell development and the few surviving pre-B cells undergo apoptosis due to a cell cycle block in G1, whereas individual KOs of these HDACs has no effect [52]. In mature B cells, the combined KO of HDAC1 and 2 has no effect on cell survival or function in the resting state, but these double KO cells fail to proliferate in response to lipopolysaccharide and IL-4 [52].

#### HATs and HDACs in T-cell development and function

HATs and HDACs also play roles in T-cell development and function. For example, the HAT p300 is important for the expression of chemokine CCR9, which is expressed in thymocytes during their migration and development into mature T cells [53]. Early in this developmental process, NOTCH signaling prevents p300 recruitment to, and acetylation of, core histones at two CCR9 enhancers, thus reducing CCR9 expression [53]. This NOTCH-dependent repression of CCR9 occurs via effects on p300 in multipotent progenitor cells and is also observed in T-lymphoma cell lines [53].

Thymus-specific deletion of the bromodomain-containing protein BRD1, which is a subunit of the HAT HBO1 complex [54], alters the pattern of CD4/CD8 expression in thymocytes and decreases the abundance of CD8+ mature T cells in the periphery [55]. Furthermore, the HBO1-BRD1 complex is responsible for activating CD8 expression by increasing global acetylation of H3K14 in developing T cells [55].

T cell-specific KO of HDAC1 does not affect late T-cell development or the number of T cells in the periphery [56]. The lack of an effect is likely due to compensation by HDAC2, whose expression is increased when HDAC1 is inactivated [56]. Moreover, T cell-specific KO of both HDAC1 and 2 results in arrested T-cell development [57], similar to what is seen in HSCs and early B cells (see above). Nevertheless, T cell-specific KO of HDAC1 alone does cause an increased Th2-type inflammatory response in a mouse model of asthma, which is characterized by elevated expression of IL-4, IL-5, and IL-10, suggesting that HDAC1 represses cytokine production in activated T cells and during T effector (Teff) cell differentiation [56]. Of note, the HDAC1-deficient increased expression of IL-4 in T cells is seen only after several rounds of cell division, suggesting that the effect of HDAC1 on IL-4 expression occurs via an epigenetic mechanism, and that the removal of repressive marks occurs during DNA replication [56]. In T cells, an HDAC1/mSIN3A complex represses IL-2 expression [58], and during T-cell activation, HDAC1-mediated repression of IL-2 is relieved by phosphorylation of mSIN3A by CDK5, which disrupts the formation of the HDAC1/mSIN3A complex [58].

HDAC6-null mice exhibit normal B-cell development, but have reduced IgM and IgG levels following antigen stimulation [59]. This defect may be due to the role of HDAC6 in immune synapse formation and T-cell migration [60, 61].

In developing T cells, the interaction of class 2 HDACs with the TF MEF2D plays a major role in regulating T-cell receptor (TCR)-mediated apoptosis during negative selection of T cells with a TCR that interacts with self-antigen. For example, HDAC7 is recruited to the NUR77 promoter by MEF2D, where it acetylates chromatin and represses the expression of this apoptotic regulator [62]. In T cells undergoing negative selection, HDAC7 or class 2 HDACs become phosphorylated near their N-termini by protein kinase D, which allows recognition by 14-3-3, disruption of the interaction with MEF2D, and results in nuclear export of the repressive HDAC [62-64]. In addition to its role in negative selection, HDAC7 regulates the expression of genes involved in positive thymic selection. For example, HDAC7 KO and mutation studies in mouse thymocytes have shown that transcription of HDAC5 is regulated by MEF2D but not by NUR77; thus, HDAC5 may be a direct target for transcriptional regulation by HDAC7 during positive, but not negative T-cell selection [65, 66]

Class 4 HDAC11 represses the expression of IL-10 in antigen-presenting cells (APCs) by interacting with the distal region of the IL-10 promoter [67]. HDAC11 localization at the IL-10 distal promoter is coupled to increased binding of the transcriptional repressor PU.1 at the distal promoter and decreased acetylation of histones H3 and H4 at the proximal IL-10 promoter [67]. APCs that overexpress HDAC11 are able to restore the responsiveness of tolerant CD4+ T cells [67].

#### HATs and HDACs in the development of T-regulatory cells

T-regulatory cells (Tregs) play an important role in limiting T-cell immune responses, and HATs and HDACs have a variety of roles in Treg function. p300 and other HATs maintain the stability and function of Tregs by acetylating the TF FOXP3, whose transcriptional output is required for Treg-mediated immunosuppression [68]. FOXP3 expression in Tregs can be either positively or negatively regulated by the TF KLF10 through its association with PCAF or SIN3-HDAC1, respectively [69]. Interestingly, p300-deficient Tregs show many of the same defects in activity, survival and proliferation that occur in FOXP3-deficient Tregs [68], suggesting that the effects of p300 deficiency on Treg function are due to a reduction in FOXP3 activity. FOXP3 has also been found in a transcriptional regulatory complex with TIP60, HDAC7, HDAC9, and other proteins [70].

The acetylation of FOXP3 by either p300 or TIP60 both protects FOXP3 from degradation and increases its DNA-binding activity, and as a result, p300- or TIP60-deficient Tregs have defects in activity, survival, and proliferation [68, 71]. Deleting either p300 or CBP in FOXP3+ Tregs in mice does not affect the overall proportion of T cells under basal conditions, and thus, these two HATs appear to have redundant roles in Treg production under resting conditions [72, 73]. When the Tregs are activated, however, p300- or CBP-deficient FOXP3+ Tregs undergo apoptosis, are unable to suppress homeostatic Teff cell proliferation, and reject transplanted allografts [72, 73]. Mice with simultaneous Treg-specific deletion of p300 and CBP develop severe autoimmunity, as both p300 and CBP interact not only with FOXP3, but also with many FOXP3-regulating TFs including NFAT, STAT1, FOXO1, FOXO3, NF-κB, RUNX1, and STAT5 [72, 74-78]. Nevertheless, p300 and CBP also have distinct roles in Tregs; for example, only p300 is required for efficient GATA-3 expression, which is important for FOXP3 expression and Treg accumulation [72].

Countering the HATs, the HDACs deacetylate FOXP3, which reduces Treg development and immunosuppressive function, and also provides a therapeutic target for enhancing immunosuppressive (and potentially anti-tumor) activity in patients [79-81]. FOXP3 can be deacetylated by certain HDACs (i.e., HDAC3, 6, 7, 9 and SIRT1), which decreases FOXP3 protein levels and activity [80, 82, 83]. Of note, HDAC6, which is normally cytoplasmic, translocates to the nucleus of some Tregs where it can deacetylate FOXP3 [81]. Treg-specific deletion of these HDACs or treatment with HDACi has been shown to enhance immunosuppressive activity and Treg function [79]. Taken together, these results suggest that acetylation of FOXP3 favors Treg development. However, while Treg development is important in limiting host autoimmunity, it may also reduce host immune responses and anti-tumor activity. Thus, inhibition of FOXP3 acetylation is a promising anti-tumor strategy [73].

### Regulation of Immune Cell-Related TFs by Acetylation

The activities of several TFs that play key roles in immune responses are affected directly and indirectly by HATs/HDACs. In most cases, the ability of CBP/p300 to acetylate a given TF and affect its activity has been investigated (summarized in Table 2). There are four general ways that direct acetylation has been shown to affect TF function: 1) lysine acetylation can increase protein stability by blocking ubiquitination of the same lysines that promote proteasome-mediated degradation; 2) lysine acetylation within the DNA-binding domain can decrease the ability of the TF to bind DNA; 3) lysine acetylation can increase (or decrease) protein-protein interactions with TF regulators; and 4) acetylated lysines on TFs can serve as a docking domain for the bromodomain of HATs, which can increase their transactivation activity [18]. For example, NF-κB and STAT subunits are important regulators of B- and T-cell development and function. Both of these TFs undergo acetylation/deacetylation at several lysines, and acetylation at different residues can positively or negatively impact their activity in distinct ways depending on the lysine residue [84, 85]. Furthermore, the activity of NF-κB and STAT can be indirectly affected by acetylation, for example, by acetylation of their specific co-activators, the TFs that interact with them, or histones at their target gene promoters. Moreover, the ability of acetylation to affect NF-κB and STAT activity can depend on the specific target gene studied.

In a small number of cases, it is known how acetylation alters the activity of a TF in a way that affects B- or T-cell function. As described above, the TF FOXP3 is a direct substrate of p300 and other HATs, and acetylation of FOXP3 plays a key role in Treg development and maintenance [68]. Acetylation increases FOXP3 activity by stabilizing the protein and enhancing its DNA-binding activity at certain promoters [68].

BCL6 is a transcriptional repressor that is essential for GC formation and lymphocyte function and proliferation [86, 87]. p300 can directly bind to and acetylate BCL6, which interferes with its ability to bind HDAC-containing complexes and consequently inactivates its repressor activity [50]. CBP/p300 mutant proteins found in some diffuse large B-cell lymphomas (DLBCLs) show a reduced ability to acetylate BCL6 [88], and therefore such lymphoma cells have increased BCL6 activity, which is related to the oncogenic state of these cells.

GATA-3 plays a key role in T-cell differentiation and survival, and acetylation has been shown to increase GATA-3 transactivation activity [89]. Moreover, overexpression of an acetylation-defective GATA3 protein affects T-cell homing to lymph nodes and increases T-cell survival after antigen stimulation [89].

### HATs and HDACs in B- and T-cell Malignancy

Given their broad role in control of lymphoid cell gene expression and TF activity, it is not surprising that misregulated acetylation is found in many cancers. As described in more detail below, in B- and T-cell cancers one often finds gene deletions and mutations that inactivate or reduce HAT activity (e.g., in CBP/p300) or overexpression of non-mutant forms of HDACs. As a consequence, reduction of global histone and TF acetylation appears to be correlated with B- and T-cell proliferation and survival, whereas increased acetylation is associated with B- and T-cell tumor growth arrest and cell death.

**Table 2:** B- and T-cell Transcription Factors That Are Regulated by Acetylation

Transcription Factor	Effect of Acetylation	Mechanism of Action	Refs
C/EBPβ	Activation	Transactivation	[155]
E2A	Activation	Transactivation, protein stability	[156, 157]
E47	Activation	Transactivation	[157]
FOXP3	Activation	DNA binding, protein stability	[68]
GATA-2	Activation	DNA binding	[158]
GATA-3	Activation	Transactivation	[89]
IRF2	Activation	Transactivation	[159]
JUN	Activation	Protein-protein interaction	[160]
MYB	Activation	Transactivation	[161]
MYC	Activation	Protein stability	[162]
NFATc1	Activation	DNA binding	[163]
NOTCH1	Activation	Protein stability	[164]
NOTCH3	Activation	Protein stability	[165]
p53	Activation	Protein stability	[166]
PAX5	Activation	Transactivation	[167]
PU.1	Activation	Transactivation	[168]
RUNX1	Activation	DNA binding/transactivation	[169]
RUNX2	Activation	Protein stability	[170]
RUNX3	Activation	Protein stability	[171]
SMAD3	Activation	Transactivation	[172]
Sp3	Activation	Transactivation	[173]
STAT3	Activation	DNA binding, transactivation; protein-protein interactions	[85]
STAT5	Activation	Dimerization, transactivation	[174]
TCF4	Activation	Protein-protein interaction	[175]
YY1	Activation	DNA binding	[176]
NF-κB	ActivationInhibition	DNA binding, IκB binding, transactivationDNA binding; IκB binding, nuclear export	[84]
BCL6	Inhibition	HDAC recruitment interference	[50]
CIITA	Inhibition	Protein degradation	[177]
ETS-1	Inhibition	DNA binding	[178]
HIF-1α	Inhibition	Protein degradation	[179]

### Mutations of HATs in B- and T-cell leukemia/lymphoma

**Figure 2:**
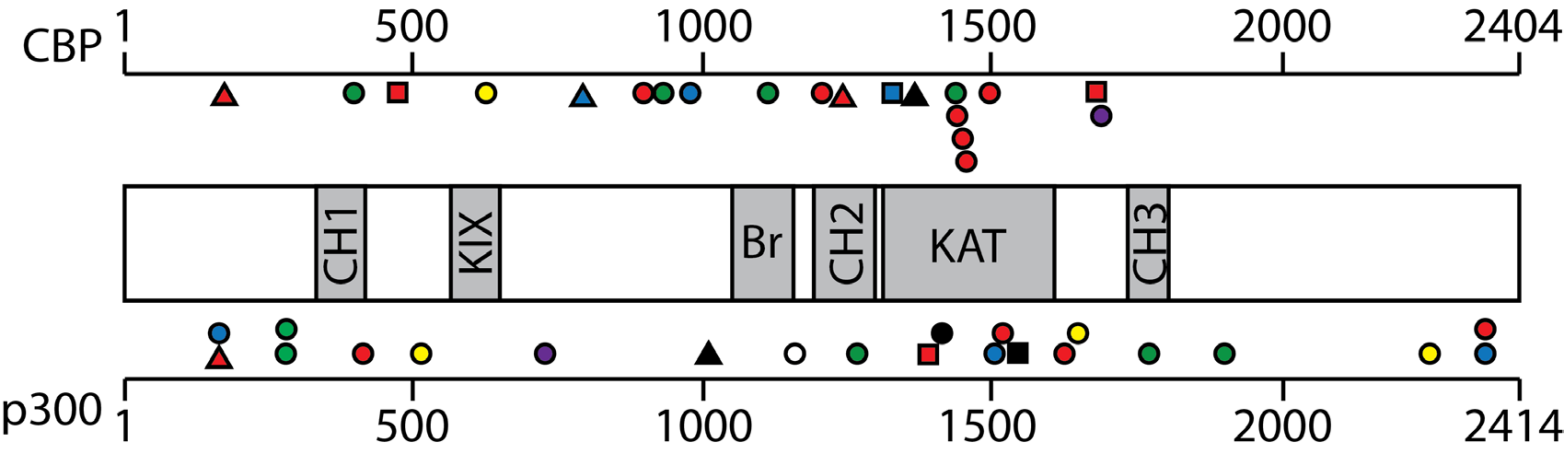
CBP/p300 mutations reported in CCLE in B- and T-cell cancer cell lines Schematic diagram of the CBP/p300 proteins with conserved domains indicated in the shaded regions as follows: cysteine/histidine domain (CH), KIX domain, bromodomain (Br), acetyltransferase domain (KAT). Symbol shapes designate types of mutations as follows: missense (circle); nonsense (triangle); and frameshift, splice site, or deletion (square). Symbol color indicates the disease type: DLBCL (red); Hodgkin's lymphoma (blue); T-cell leukemia (acute lymphoblastic or anaplastic large cell) (green); plasma cell myeloma (yellow); acute lymphoblastic B-cell leukemia (purple); B-cell lymphoma unspecified (black); and Burkitt's lymphoma (white). CBP mutations are (in order, left to right) Q170*, M395T, L470fs, A620V, Q790*, P901L, P928A, P975L, S1108L, K1203 splice, E1238*, T1332I, R1360*, S1432P, D1435E, F1440L, R1446L, Q1491K, S1680del, and S1687P. p300 mutations are Q160*, M165I, V279I, S281T, L415P, M514V, R728W, E1011*, E1160V, A1268V, R1391 splice, H1415P, G1506V, L1520V, K1546fs, R1627W, S1650F, R1773W, Q1904P, A2259T, P2358L, P2367L.

Although chromosomal translocations involving p300, CBP and MYST are well-documented in acute myeloid leukemia [90], they have not been found in B- and T-cell malignancies. However, other types of HAT gene mutations are common in certain types of B- and T-cell cancers. Namely, the genes encoding CBP and p300 harbor point mutations or deletions in approximately 20–40% of DLBCL [88, 91, 92], about 70% of follicular lymphomas (FL) [93], and less frequently in T-cell leukemia, acute lymphoblastic leukemia (ALL) and myelodysplastic syndrome [94, 95]. The TIP60 gene frequently suffers mono-allelic loss and reduced expressed in several types of B-cell lymphoma [95]. Moreover, our analysis of the Cancer Cell Line Encyclopedia (CCLE) database [96] finds that mutations in CBP/p300 and other HATs (especially MORF) are common in a variety of B- and T-cell cancer cell lines (Table 3). With CBP and p300, the majority of these lymphoma mutations occur within or near the HAT domain or introduce frame-shifts or stop codons N-terminal to the HAT domain (see Figure 2). Thus, many of the CBP/p300 mutations found in DLBCL and FL are predicted to reduce acetyltransferase activity [88]. Indeed, several of these point mutations have been demonstrated to impair the affinity of CBP for acetyl-CoA and consequently compromise the ability of CBP to acetylate the TFs BCL6 and p53 [88]. Of note, acetylation of BCL6 decreases its gene repressing activity, whereas acetylation of p53 is required for its gene activation function (Table 2) [50, 97]. Thus, DLBCL cells with HAT gene mutations have higher levels of active BCL6 and lower levels of active p53 [88], consistent with decreased acetylation being associated with increased tumor cell growth.

In contrast to the more common point mutations, genomic alterations that completely remove the HAT domain in CBP or p300 are present in a minority of DLBCL and FL tumors and cell lines [88, 93]. Expression of C-terminally truncated CBP/p300 proteins missing the HAT domain has been demonstrated in some DLBCL cell lines [88, 98, 99]. A variety of evidence suggests that these HAT-deficient p300 mutants play an active role in lymphomagenesis. First, expression of the HAT-deficient p300 proteins is preferentially retained in these cell lines, whereas the wild-type allele is silenced [98, 99]. Second, knockdown of p300 mutant protein expression reduces the growth of some DLBCL cell lines [98, 99]. Third, HAT-deficient p300 mutants localize to sites of active transcription in cell nuclei, interact with the lymphomagenic TF REL, and affect transcription of REL target genes [98-100]. Moreover, expression of a HAT-deficient mutant of p300 increases the proliferation of HSCs that lack wild-type p300 [27]. DLBCL cell lines that express HAT-defective p300 mutants have generally lower levels of histone H3 acetylation at K14 and K18 [99]. Low levels of H3K14 and H3K18 acetylation have been associated with proliferation in other cell types [101], and CBP/p300 catalyze nearly all H3K18 acetylation in mice [102]. Thus, even though they are defective for HAT activity, C-terminally truncated p300 proteins appear to contribute to B-cell transformation, at least in part, by acting as aberrant scaffolds that organize altered transcription complexes at a variety of gene promoters/enhancers to cause a broad-range of transcriptional deregulation. For example, we have previously suggested that dampening of global REL/NF-κB-dependent gene transcription is one oncogenic effect of p300 mutants in DLBCL [99].

Similar to the effects of mutations on HAT activity in lymphoma, the HBZ protein of Human T-cell Leukemia Virus type 1 (HTLV-1) binds to and inactivates the HAT domains of CBP and p300, and consequently reduces cellular levels of H3K18 acetylation [103, 104]. Thus, inhibition of CBP/p300 HAT activity may also be important for HTLV1-induced T-cell leukemia.

Interestingly, the TF BCL6, which is upregulated in and required for the growth of approximately 70% of DLBCLs [105], appears to be a direct transcriptional repressor of the p300 gene [106]. Furthermore, induced expression of p300 is required for the anti-proliferative effects of BCL6 inhibitors on DLBCL cell lines [106]. Consequently, DLBCL cell lines with defective p300 proteins are resistant to the anti-growth effects of BCL6 inhibitors, and in these cell lines, HDACi synergize with BCL6 inhibitors for inhibition of DLBCL cell growth [106].

**Table 3:** HAT Gene Mutations in B- and T-cell Malignancies

HAT	Percent of B- and T- cell lines with HAT gene mutations from CCLE [96]	Percent of HAT gene mutations in leukemia/lymphoma from select genome-wide studies
ATF2	0.6	
CBP	13.3	DLBCL, 19% (443) [88, 91-93, 180, 181] FL, 50% (52) [92, 93], Relapsed ALL, 18% (71) [182]
p300	10.5	DLBCL, 11% (546) [88, 91-93, 106, 180] FL, 19% (52) [92, 93]
TIP60	0	DLBCL, 2% (49) [180]
MOZ	7.7	DLBCL, 4% (53) [181]
MORF	27.6	
HBO1	0.6	
NCOA1	2.2	
NCOA2	3.9	DLBCL, 2% (53) [181]
NCOA3	39.8	DLBCL, 4% (53) [181]
CLOCK	0	DLBCL, 2% (102) [180, 181]
TAF1	3.3	FL, 10% (39) [93] DLBCL, 5% (102) [180, 181]

Percentage of cell lines mutated in CCLE (Cancer Cell Line Encyclopedia) indicates the percentage of 181 hematopoietic and lymphoid cell lines that had a mutation in the indicated HAT gene. DLBCL and FL genomic studies indicate the percentage of cases reported to have mutations in the indicated HAT gene in either DLBCL (diffuse large B-cell lymphoma) or FL (follicular lymphoma) patient samples and/or cell lines, with the total number of samples analyzed shown in parentheses.

Overall, there are no good mouse models for HAT gene mutations in B- and T-cell malignancy. In one report [107], a single mouse reconstituted with CBP-null HSCs developed a thymic lymphoma that arose from the CBP-null cells, but that mouse has not been further characterized. Based on the inactivation of the wild-type *EP300* allele in DLBCLs containing certain p300 mutations [98, 99], expression of truncated or mutant p300 proteins (from human DLBCLs) in p300−/− B-cell precursors may lead to B-cell malignancy in a transgenic mouse model.

### HDAC dysregulation in B- and T-cell lymphoma/leukemia

Unlike the case with HATs, mutations in genes encoding HDACs have not been found in any B- and T-cell malignancies. However, HDACs have been reported to have altered (usually increased) expression in a variety of B- and T-cell malignancies, including DLBCL, FL, and chronic lymphocytic leukemia (CLL) (Table 4). For example, HDAC1 is overexpressed in some T-cell lymphomas [108-112], while HDAC6 has been reported to be both overexpressed [110, 113] and underexpressed [114] in DLBCL.

At this point, two of the most relevant questions are whether altered expression of a specific HDAC contributes to the growth or survival of the tumor cells (and how it does so) and whether altered HDAC expression can be prognostic for therapy. In a smattering of cases, there are data addressing these questions, but the overall picture is still not clear. Inhibition of HDAC8 induces apoptosis in T cell-derived lymphoma and leukemic cells, but not in solid tumors [115]. High HDAC4 expression is associated with a poor response to prednisone in ALL, and siRNA-mediated inhibition of HDAC4 has been shown to sensitize a T-ALL cell line to etoposide-induced cell death [116]. Moreover, the interaction of HDAC4 with the leukemic PLZF-RARα fusion protein contributes to oncogenesis because it is required for the repression of differentiation-associated genes [117].

In childhood acute lymphoblastic leukemia (ALL), high HDAC3 expression has been associated with a better prognosis, whereas overexpression of HDAC7 and 9 have been associated with a poorer prognosis [118]. A study of over 200 adult CLL B-cell tissue samples reported that overexpression of HDAC7 and 10 and underexpression of HDAC6 and SIRT3 are correlated with a poor prognosis [119]. HDAC6 overexpression correlates with a more favorable outcome in DLBCL, but with a negative outcome in peripheral T-cell lymphoma [110]. Although HDAC1, 2 and 3 are all overexpressed in Hodgkin's lymphoma tissue samples, only high HDAC1 expression is correlated with a worse outcome [120].

**Table 4:** Misregulated Expression of HDACs in B- and T-cell Malignancies

HDAC	Disease	Expression	Refs
1	T-ALL	Increased	[118]
	B-ALL	Increased	[119]
	ALL	Increased	[116]
	HL	Increased	[120]
	DLBCL	Increased	[110]
	CLL	Increased	[183]
2	ALL	Increased	[116, 118]
	B-ALL	Reduced	[119]
	HL	Increased	[120]
	DLBCL	Increased	[110]
3	ALL	Increased	[118]
	CLL	Increased	[183]
	DLBCL	Increased	[184]
	HL	Increased	[120]
4	T-ALL	Increased	[118]
5	B-ALL	Increased	[118]
6	ALL	Increased	[118]
	B-ALL	Increased	[119]
	CLL	Increased	[183]
	DLBCL	Increased	[110]
7	ALL	Increased	[118]
	B-ALL	Increased	[119]
	CLL	Increased	[183]
8	ALL	Increased	[116, 118]
9	CLL	Increased	[183]
10	CLL	Increased	[183]
11	B-ALL	Reduced	[119]
	B-ALL	Increased	[119]
SIRT1	CLL	Increased	[183]
SIRT3	B-ALL	Increased	[119]
SIRT4	B-ALL	Decreased	[119]
SIRT5	B-ALL	Decreased	[119]
SIRT6	B-ALL	Increased	[119]
	CLL	Increased	[183]
SIRT11	B-ALL	Increased	[119]

ALL, acute lymphocytic leukemia; CLL, chronic lymphocytic leukemia; DLBCL, diffuse large B-cell lymphoma; HL, Hodgkin's lymphoma.

As with HATs, there are no good mouse models for the role of HDACs in cancer. Based on most evidence, it is unlikely that overexpression of any HDAC would, by itself, be oncogenic. Thus, one method for evaluating the molecular mechanisms by which increased HDAC expression contributes to oncogenesis might be to create transgenic mice with B and/or T cell-specific expression of a relevant HDAC (e.g., HDAC6) and cross such mice to other common transgenic mouse tumor models (e.g., Eμ-MYC mice). One could then determine whether increased HDAC expression leads to enhanced tumor development or if such mice develop chemo- or HDACi-resistant tumors.

### HAT and HDAC Inhibitors in the Treatment of B- and T-cell Cancers

Given that mutations and dysregulation of HATs and HDACs occur in many B- and T-cell cancers, as well as their global effects on protein activity and gene expression, these enzymes have been investigated for therapeutic targeting. Below we discuss the types of compounds that have been found to inhibit HAT and HDAC activity, and examples of such molecules being used in the treatment of lymphoid cancer cells. Overall, HDACi have been more useful in such settings than HATi, and HDACi are being used in the clinic to treat lymphoid cell cancers.

### HAT inhibitors (HATi)

Several types of compounds have been characterized as HAT inhibitors, including a variety of synthetic compounds and natural products and their derivatives. In general, such compounds are pan-HAT inhibitors or inhibitors of GCN5 or CBP/p300 [121, 122].

There are few reports of HATi as inhibitors of B- or T-cell cancers, and no HATi are currently FDA approved. Anacardic acid, found in cashew nuts, is a potent inhibitor of p300, PCAF, and TIP60 [123, 124]. Anacardic acid and derivatives have been shown to inhibit Jurkat T-cell leukemia cells at micromolar concentrations [125]. Of note, Jurkat cells have been shown to express two TIP60 variants, including one with a deleted HAT domain [126]. The natural products gallic acid and curcumin have both been shown to act as HAT inhibitors [127, 128], and can induce proliferation arrest and apoptosis in lymphoma cells [129, 130]. However, gallic acid and curcumin are not especially potent inhibitors of lymphoma cell growth and both have many protein targets [131, 132]; thus, it is not clear that their effects on lymphoma cell growth are due to their anti-HAT activity. The synthetic compound C646 is a specific p300 inhibitor, however, it was not especially effective against leukemia cell lines in a screen of the National Cancer Institute (NCI) 60-cell line panel [133].

### HDAC inhibitors (HDACi)

Given that increased HDAC expression and activity is found is many lymphoid malignancies (see above), it is perhaps not surprising that HDACs should emerge as targets for therapy. In contrast to HATi, HDACi have been extensively studied for anti-cancer activity. Indeed, since 2001, a number of HDACi have been used in the clinic for the treatment of various cancers, including B- and T-cell cancers [134]. Alone or in combination with other anti-cancer agents, a variety of HDACi have been shown to induce apoptosis in many different types of B- and T-cell lymphoma and leukemia cell lines (Table 5). Following from those studies, several HDACi have been tested clinically for the treatment of such human cancers, including cutaneous T-cell lymphoma (CTCL), DLBCL, multiple myeloma (MM), FL, Hodgkin's lymphoma (HL), and several others (Table 6), and as of April 2015, there are at least 12 ongoing clinical trials testing HDACi alone or in combination with other cancer therapeutics for the treatment of several B- and T-cell malignancies (Table 7).

HDACi fall into five main classes, based in part on their chemical structures and in part on their specificity. These include the following: 1) hydroxyamic acids, 2) cyclic tetrapeptides, 3) benzamides, 4) ketones, and 5) aliphatic acids. In addition, HDACi can have broad-based pan-HDAC inhibitory activity, have class specificity, or even isozyme specificity. Currently, four HDACi have received FDA approval for clinical use. The first two FDA-approved inhibitors are the pan-HDACi vorinostat (aka suberoylanilide hydroxamic acid [SAHA]), which is available as an oral medication, and the class I HDACi romidepsin (a bacterial cyclic peptide), which is administered intravenously. HDACi treatment is especially effective in the treatment of CTCL, with favorable response rates (from a number of trials) of approximately 70% when using romidepsin (Table 6). Although it is not known why CTLC responds well to HDACi treatment, increased expression of HDAC2 and histone H4 acetylation have been correlated with aggressive CTCL [110]. The HDACi belinostat was approved in 2014 for relapsed and refractory peripheral T-cell lymphomas [135]. Finally, the HDACi panobinostat, another hydroxamate, has been approved by the FDA for refractory multiple myeloma [136]. Because of the role of HDACs in normal immune cell function, one problem with using HDACi treatment for anti-cancer therapy is the concomitant suppression of host immune responses required for anti-tumor therapy (see also *HATs and HDACs in development of T-regulatory cells* section above) [137].

In the simplest scenario, HDACi treatment increases the level of acetylation of histones on chromatin, thereby increasing gene expression, and HDACi also increase the acetylation of non-histone proteins. For lymphoid cell TFs, increased acetylation can either increase or decrease their activity (Table 2). Because of the myriad effects of acetylation/deacetylation on gene expression and protein activity, it is almost certain that the effects of HDACi on tumor cell growth and survival are not through single or even a small number of targets. Moreover, the effects of HDACi would be expected to vary among tumor cell types, within a given tumor type, and, due to tumor cell heterogeneity, even within a given tumor.

**Table 5:** HDACi Compounds That Induce Apoptosis in B- and T-cell Cancer Cells

HDACi	Class	Target of HDACi	Clinical trial stage	Hematopoietic malignancy (Patient and Cell Line)	Combination treatments that induce apoptosis	Refs
Butyrate (NaB)	Short-chain fatty acid	Class 1, 2a	Phase I, II	B-lymphoma, BL	Cisplatin, Etoposide	[142, 185, 186]
Valproic Acid (VPA)	Short-chain fatty acid	HDAC1-5, 7, 8, 10	Phase I, II, III	AML, CML, CLL, DLBCL, HL, MM, NHL, NK cell lymphoma, SLL, T-cell lymphoma	5-Azacytidine, ATRA, Bortezomib, Cambinol, Cyclophosphamide, Decitabine, Enzastaurin, Etoposide, EX527, Imatinib, Pioglitazone, Prednisone, Rituximab, Sirtinol, Temozolomide, Vincristine	[142, 187-193]
Tricostatin A (TSA)	Hydroxamate	Class 1, 2, 4		AML, B-lymphoma, DLBCL, EBV+ BL, NHL	Decitabine	[142, 146, 186, 194-197]
Vorinostat, Suberoylanilide hydroxamic acid (SAHA)	Hydroxamate	Class 1, 2, 4	Phase I, II, III	ALL, AML, CLL, CML, CTCL, DLBCL, HL, MCL, MM, NHL	17-AAG, ABT-737, Azacitidine, Bexarotene, Bortezomib, Carboplatin, Carfilzomib, Cladribine, Cyclophosphamide, Decitadine, Eltrombopag, Enzastaurin, Etoposide, Ifosfamide, Lenalidomide, Melphalan, NPI-0052, Prednisone, Rituximab	[67, 134, 142, 146, 198-209]
Belinostat (PDX101)	Hydroxamate	Class 1, 2, 4	Phase I, II, III	ALL, AML, CLL, MM, MCL, NHL, PTCL, T-cell lymphoma	17-AAG, Azacitidine, Bortezomib	[142, 188, 206, 210-214]
Dacinostat (LAQ824)	Hydroxamate	Class 1, 2, 4	Phase I	Acute eukemia, AML progenitor cells, CLL, meyloid leukemias	Decitabine	[142, 215-219]
Panobinostat (LBH589)	Hydroxamate	Class 1, 2, 4	Phase I, II, III	AML, CLL, CML, CTCL, DLBCL, HL, MCL, MM, NHL, NK/T-cell lymphoma, PTCL	17-AAG, Bortezomib, Carboplatin, Cytarabine, Etoposide, Everolimus, Idarubicin, Imatinib, Ifosfamide, Lenalidomide, Pemetrexed, Rituximab	[134, 142, 203, 220-224]
Suberic bishydroxamic acid (SBHA)	Hydroxamate	Class 1, 2, 4		ALL, Leukemia, MM	ABT-737	[142, 145, 225, 226]
Azelaic bishydroxamic acid (ABHA)	Hydroxamate	Class 1, 2, 4		EBV+ B-cell lines	ABT-737	[142, 227]
SK-7041	Hydroxamate	HDAC1, 2		Meyloid leukemia	Imatinib	[228, 229]
ITF-A and ITF-B	Hydroxamate	Class 1, 2, 4	Phase I	DLBCL, MCL, SMZL		[151]
Tubacin	Hydroxamate	Class 2b		ALL, AML, CML, EBV+ BL, MM	17-AAG, Bortezomib	[142, 230-233]
JNJ 26481585	Hydroxamate	Class 1, 2, 4	Phase I	CTCL, Leukemia, MM	Bortezomib, Dexamethasone	[234]
PCI-24781	Hydroxamate	Class 1, 2, 4	Phase I	HL, NHL	Bortezomib, Pazopanib	[235, 236]
Entinostat (MS-275)	Benzamide	HDAC1-3	Phase I, II	ALL, AML, CML, HL, MM	Azacitadine, Imatinib, Isotretinoin, Sargramostim, Sorafenib, Rapamycin, Rituximab	[142, 237-241]
Mocetinostat (MGCD-0103)	Benzamide	HDAC1-3, 10, 11	Phase I, II	AML, CLL HL, NHL	5-azacitadine, Bortezomib, Docetaxel, Gemcitabine, GX15-070	[242-250]
Romidepsin (FK228)	Cyclic tetrapeptide	Class 1, 2, 4	Phase I, II, III	ALL, AML, CLL, CTCL, DLBCL, MM, MCL, NHL, PTCL, SLL	Bortezomib, Carboplatin, Cyclophosphamide, Decitadine, Etoposide, Ifosfamide, Prednisone, Rituximab, Vincrstine	[206, 210, 223, 251-260]
Apicidin	Cyclic tetrapeptide	HDAC1, 3		APL, CML	Imatinib, TRAIL	[142, 261-264]
Nicotinamide	Vitamin B member	Sirtuins		CLL		[265]
Tenovin-6 (TV-6)	Small Molecule	SIRT1		CML	Imatinib	[266]
Amurensin G	Natural Product	SIRT1		TRAIL-resistant leukemia		[267]

Cell types: ALL, acute lymphoblastic leukemia; AML, acute myeloid leukemia; APL, acute promyelocytic leukemia; BL, Burkitt's lymphoma; CLL, chronic lymphocytic leukemia; CML, chronic myelogenous leukemia; CTCL, cutaneous T-cell lymphoma; DLBCL, diffuse large B-cell lymphoma; EBV+ BL, Epstein-Barr virus-positive BL; HL, Hodgkin's lymphoma; MCL, mantle cell lymphoma; MM, multiple myeloma; NHL, non-Hodgkin's lymphoma; PTCL, peripheral T-cell lymphoma; SLL, small lymphocytic lymphoma.

Drug type: 17-AAG (Hsp90 inhibitor); ABT-737 (BH3-mimetic); ATRA (all-trans retinoic acid); azacitidine (DNA methyltransferase inhibitor); bexarotene (antineoplastic agent); bortezomib (proteasome inhibitor); cambinol (sirtuin inhibitor); carboplatin (antineoplastic agent); carfilzomib (proteasome inhibitor); cisplatin (alkylating agent); cladribine (adenosine deaminase inhibitor); cyclophosphamide (alkylating agent); cytarabine (DNA synthesis inhibitor); decitabine (DNA methyltransferase inhibitor); dexamethasone (glucocorticoid steroid); docetaxel (anti-mitotic agent); eltrombopag (thrombopoeitin receptor agonist); enzastaurin (PKCβ inhibitor); etoposide (topoisomerase inhibitor); everolimus (mTOR inhibitor); EX527 (sirtuin inhibitor); gemcitabine (nucleoside analog); GX15-070 (BH3-mimetic); idarubicin (topoisomerase II inhibitor); imatinib (tyrosine kinase inhibitor); ifosfamide (alkylating agent); isotretinoin (retinoic acid analog); lenalidomide (tumor necrosis factor [TNF] inhibitor); melphalan (alkylating agent); NPI-0052 (proteasome inhibitor); pazopanib (tyrosine kinase inhibitor); pemetrexed (folate antimetabolites); pioglitazone (thiazolidinedione); prednisone (glucocorticoid prodrug); rituximab (anti-CD20 antibody); sargramostin (recominant GM-CSF); sirtinol (sirtuin inhibitor); sorafenib (tyrosine kinase inhibitor); temozolomide (alkylating agent); TRAIL (TNF-related apoptosis-inducing ligand ); vincristine (mitotic inhibitor).

Consistent with those hypotheses, treatment of CTCL cell lines with vorinostat showed that HDACi treatment leads to hyperacetylation of all core histones, which is associated with changes in the expression of genes involved in regulation of the G1/S and G2/M transitions, apoptosis, anti-proliferation, and MAPK signaling [138]. Overall, gene expression profiling showed that up to 22% of genes are altered by HDACi as early as four hours post treatment in several cell types [139-141]. Nevertheless, there does appear to be a common set of genes that change expression in response to HDACi treatment, and these genes include several cyclins, the cell-cycle inhibitor p21, p53, BAX, BCL2, MYC, PKCδ, ICAM-1, IL-6 receptor, IL-2, IL-8, IL-10, VEGF, NOTCH, GADD45 and GADD45, TGF receptor, CTP synthase, and TYMS (reviewed by [17, 134, 142]. At least in part, HDACi induce cell-cycle arrest by causing accumulation of hyperacetylated p53, which then induces expression of p21, leading to inhibition of cyclins D and A, which are required for cell-cycle progression (reviewed by [134, 142]). However, because CTCL tumors typically grow quite slowly in patients, it is unclear how the reported effects of HDACi on cell-cycle progression in rapidly growing cell lines *in vitro* reflect its effects on CTCL tumors *in vivo*.

HDACi treatment of tumor cells has been frequently linked to the modulation of BCL2 family expression to favor a pro-apoptotic expression pattern (reviewed by [134, 142, 143]). In many cases, HDACi-induced apoptosis occurs via increased expression of pro-apoptotic BCL2 family members BIM, BAX, PUMA, and NOXA (reviewed by [142, 144, 145]). Moreover, resistance to HDACi-induced apoptosis can be achieved in DLBCL cells lines by artificial or induced expression of anti-apoptotic protein BCL-XL [146]. Increased activity of BCL2, thioredoxin, and CHK1 has also been associated with the development of HDACi resistance in lymphoma [147].

**Table 6:** Summary of Published Clinical Trials of FDA-approved HDACi in Lymphoma, Leukemia, and Myeloma

HDACi	Trial phase	Combination drug	Cancer type	No. of patients	CR	PR	SD	% Response	Refs
Vorinostat	I		DLBCL, HL, MM, T-cell lymphoma, MCL, SLL, ML	35	1	4	3	23	[268]
	I		FL, MCL, DLBCL, CTCL	10	3	1	3	70	[269]
	I	Idarubicin	Relapsed or refractory leukemia	41	3	10		32	[270]
	I		Advanced MM	13		1	9	77	[271]
	I	Bortezomib	Relapsed or refractory MM	21		9		43	[272]
	I		Advanced leukemias, myelodysplastic syndromes (MDS)	41	4	3		17	[273]
	I		Advanced CTCL	74		22		30	[204]
	I		HL, NHL, DLBCL, SLL, MCL, CTCL, PTCL, Myeloma, AML, MDS	23	1	1	4	26	[274]
	II	Idarubicin, Cytarabine	AML, MDS	75	57	7		85	[275]
	II	Gemtuzumab ozogamicin	AML	31	6	1		23	[276]
	II		Relapsed or refractory HD	25		1	7	32	[277]
	II		NHL, MCL, relapsed or refractory FL, MZL	35	5	5	1	31	[278]
	II		Refractory CTCL	33		8	1	27	[279]
	IIb		Advanced CTCL	6		5	1	100	[280]
	IIb		Refractory CTCL	74		22		30	[281]
		Bortezomib	Relapsed or refractory MM	6		5	1	100	[282]
			**TOTALS**	**543**	**80**	**105**	**30**		
Romidepsin	I		CTCL	4	1	3		100	[258]
	II		PTCL	130	33	14	33	62	[283]
	II	Low-dose beam radiation	CTCL	5		4		80	[284]
	II		PTCL	45	8	9	5	49	[256]
	II		Refractory CTCL	96	6	27	45	81	[260]
	II		CTCL	71	4	20	26	70	[257]
			**TOTALS**	**351**	**52**	**77**	**109**		
Panobinostat	I		CTCL	10	2	4	1		[221]
	I	Ifosfamide, Carboplatin, Etoposide	Relapsed or refractory cHL	21				86	[285]
	I	Everolimus	Relapsed or refractory lymphoma	30	3	7		33	[286]
	Ib	Bortezomib	Relapsed or refractory MM	62	2	34		61	[287]
	Ia/II		Refractory HL	13		13			[220]
	I/II	Melphalan	Relapsed or refractory MM	40		3	23		[288]
	II		Relapsed or refractory MM	38		2	9	5	[289]
	II	Bortezomib, Dexamethasone	Relapsed or refractory myeloma	55	1	28	8	35	[290]
	II		Refractory CTCL	139	2	22	29	17	[291]
	II		Relapsed or refractory HL	129	5	30	71	27	[292]
	II	Melphalan, Thalidomide, Prednisone	Relapsed or refractory MM	31	2	10	11	39	[293]
	III	Bortezomib, Dexamethasone	Relapsed or refractory MM	387	42	216	65	67	[136]
			**TOTALS**	**955**	**59**	**369**	**217**		
Belinostat	I		NHL, HL, MM, CLL	16			5		[213]
	II		Relapsed or refractory CTCL	29	3	1	10	4	[294]
	II		Recurrent PTCL	24	2	4	4	6	[294]
	III		Relapsed or refractory CTCL	120	11	15		26	[135]
			**TOTALS**	**189**	**16**	**20**	**19**		

CR, complete response; PR, partial response; SD, stable disease.

**Table 7:** Ongoing clinical trials using HDACi for treatment of lymphoma, leukemia, and myeloma

Trial title	HDACi	Combination target	Phase
WEE1 Inhibitor MK-1775 and Belinostat in Treating Patients With Relapsed or Refractory Myeloid Malignancies or Untreated Acute Myeloid Leukemia	Belinostat	WEE1	I
Belinostat and Yttrium Y 90 Ibritumomab Tiuxetan in Patients W/Relapsed Aggressive B-Cell NHL	Belinostat	Radiotherapy	II
Panobinostat and Everolimus in Treating Patients With Recurrent Multiple Myeloma, Non-Hodgkin Lymphoma, or Hodgkin Lymphoma	Panobinostat	mTOR	I, II
Panobinostat in Treating Patients With Relapsed or Refractory Non-Hodgkin Lymphoma	Panobinostat		II
Romidepsin in Treating Patients With Lymphoma, Chronic Lymphocytic Leukemia, or Solid Tumors With Liver Dysfunction	Romidepsin		I
Alisertib and Romidepsin in Treating Patients With Relapsed or Refractory B-Cell or T-Cell Lymphomas	Romidepsin	Aurora Kinase	I
Rituximab, Romidepsin, and Lenalidomide in Treating Patients With Recurrent or Refractory B-cell Non-Hodgkin Lymphoma	Romidepsin	CD20, immunomodulation, proliferation, angiogenesis	I, II
Romidepsin and Lenalidomide in Treating Patients With Previously Untreated Peripheral T-Cell Lymphoma	Romidepsin	Immunomodulation, proliferation, angiogenesis	II
Alisertib in Combination With Vorinostat in Treating Patients With Relapsed or Recurrent Hodgkin Lymphoma, B-Cell Non-Hodgkin Lymphoma, or Peripheral T-Cell Lymphoma	Vorinostat	Aurora Kinase	I
Vorinostat and Combination Chemotherapy With Rituximab in Treating Patients With HIV-Related Diffuse Large B-Cell Non-Hodgkin Lymphoma or Other Aggressive B-Cell Lymphomas	Vorinostat	CD20	I, II
Bortezomib and Vorinostat as Maintenance Therapy After Autologous Stem Cell Transplant in Treating Patients With Non-Hodgkin Lymphoma	Vorinostat	Proteosome	II
Cytarabine and Daunorubicin Hydrochloride or Idarubicin and Cytarabine With or Without Vorinostat in Treating Younger Patients With Previously Untreated Acute Myeloid Leukemia	Vorinostat	DNA synthesis, Topoisomerase II	III

There is considerable interest in identifying markers that can predict responsiveness to HDACi therapy [148]. Markers that have been reported to predict better response to HDACi treatment include high levels of shuttling protein HR23B [149] and several induced mRNAs, including cyclin D1 [150] for CTCL and CDKN1A [151] for DLBCL. Interestingly, it has been reported that DLBCLs with mutations in p300 or CBP are more responsive to HDACi treatment [152-154], suggesting that decreased HAT activity makes HDACi treatment more successful and that combined treatment with HATi and HDACi could be a useful strategy.

One note of caution in the use of HDACi is the finding that loss of HDAC1/2 activity by gene KO in mouse T cells has been reported to lead to T-cell malignancy, and these malignant cells show increased expression of the oncoprotein MYC and aneuploidy [57].

## CONCLUSIONS AND PERSPECTIVES

The role of acetylation in regulating chromatin structure, gene expression, and protein activity will undoubtedly continue to receive much attention. Given the complex signaling and gene expression changes that occur in B- and T-cell development, there is much more to be learned about the role of regulated acetylation in these processes.

Although the use of HATi for therapy is at an early stage, HDACi treatment is likely to continue for the treatment of B- and T-cell malignancies and certain immune diseases. Thus, a deeper understanding of the proteins, genes, and pathways affected by deregulated acetylation will be crucial to applying HDACi in the clinic. Given the wide range of transcriptional regulators affected by acetylation, there are clearly many targets affected by HDACi treatment and these targets no doubt vary among different cancers. Thus, HDACi may be most effective when combined with therapeutics that target specific pathways in individual cancers. The use of HDACi in combination with other therapeutics is a strategy that is being used in many ongoing clinical trials (Table 7), and the ability to prescribe appropriate combined HDACi-targeted drug regimens will improve as better ways are developed to molecularly profile pathways that are driving individual cancers.
